# Combining naproxen and a dual amylin and calcitonin receptor agonist improves pain and structural outcomes in the collagen-induced arthritis rat model

**DOI:** 10.1186/s13075-019-1819-9

**Published:** 2019-02-22

**Authors:** Anna Katri, Aneta Dąbrowska, Henrik Löfvall, Ming Ding, Morten A. Karsdal, Kim V. Andreassen, Christian S. Thudium, Kim Henriksen

**Affiliations:** 1 Department of Drug Design and Pharmacology, University of Copenhagen, Copenhagen, Denmark; 2 Biomarkers and Research, Nordic Bioscience, Hovedgade 205-207, 2730 Herlev, Denmark; 3 Division of Molecular Medicine and Gene Therapy, Lund Strategic Center for Stem Cell Biology, Lund, Sweden; 4 Department of Orthopaedics and Traumatology, Institute of Clinical Research, Odense University Hospital, University of Southern Denmark, Odense, Denmark

**Keywords:** Rheumatoid arthritis, CIA, DACRA, NSAIDs, Pain, Bone, Treatment

## Abstract

**Background:**

Pain is a debilitating symptom of rheumatoid arthritis (RA), caused by joint inflammation and cartilage and bone destruction. Nonsteroidal anti-inflammatory drugs (NSAIDs) are used to treat pain and inflammation in RA, but are not disease-modifying and do not prevent joint destruction when administered alone. KBPs (Key Bioscience peptides) are synthetic peptides based on salmon calcitonin and are expected to inhibit bone resorption and to be chondroprotective. In this study, we investigated if combining a standard of care NSAID (naproxen) with a KBP resulted in improvement in pain scores, as well as disease activity and structural damage in a rat model of RA.

**Methods:**

Collagen-induced arthritis (CIA) was induced in 40 female Lewis rats by immunization with porcine type II collagen; 10 rats were given sham injections. CIA rats were treated with KBP and/or naproxen. Health scores and joint scores were evaluated daily. Mechanical and cold allodynia tests and burrowing tests were used to assess pain-like behaviors. Blood samples were collected for biomarker testing, and paws were collected for histology and microcomputed tomography.

**Results:**

Naproxen monotherapy increased the time until humane endpoints was reached, and improved health score, pain assessments, and trabecular thickness, while KBP monotherapy did not result in improvements. Combination therapy had improved efficacy over naproxen monotherapy; combination therapy resulted in improved health scores, and importantly reduced mechanical and cold allodynia assessment. Furthermore, protection of articular cartilage structure and preservation of bone structure and bone volume were also observed.

**Conclusions:**

This study demonstrates that combining KBP and naproxen may be a relevant therapeutic strategy for RA, resulting in improvements to the overall health, pain, inflammation, and joint structure.

**Electronic supplementary material:**

The online version of this article (10.1186/s13075-019-1819-9) contains supplementary material, which is available to authorized users.

## Background

Rheumatoid arthritis (RA) is a chronic autoimmune-mediated disease of the joint, associated with infiltration of immune cells such as lymphocytes, synovial lining hyperplasia, and bone and cartilage destruction, which manifests as pain leading to loss of joint function, physical impairment, and fatigue [1]. RA is associated with continuous bone loss and bone erosion as the normal interplay between osteoblasts and osteoclasts is disturbed [2], often leading to increased fracture risk and osteoporosis [3–5]. The inflammatory processes are responsible for the pain outcome [6] as well as activation of peripheral and central neuronal mechanisms [7]. As such, 70% of the RA patients express a yearning for more effective pain relief [8, 9].

Current standard of care for RA includes disease-modifying antirheumatic drugs (DMARDS), analgesics, nonsteroidal anti-inflammatory drugs (NSAIDs), and corticosteroids. The most effective RA treatments are the DMARDS, including methotrexate, and inhibitors of tumor necrosis factor (TNF) and IL-6. Analgesics and NSAIDs are used for symptomatic relief, but are not disease-modifying [10]. Current RA treatments come with several, potentially severe, side effects including immunosuppression, infections, osteoporosis, gastrointestinal bleeding, liver toxicity, and pulmonary fibrosis, leading to reduced quality of life and high risk of hospitalization and mortality [11–13]. Thus, novel treatments with a more benign safety profile are needed.

Calcitonin (CT) is a 32-amino acid peptide hormone which is secreted from the parafollicular cells of the thyroid gland in response to increased serum calcium concentration. Salmon calcitonin (sCT)—along with other teleost and avian calcitonins, e.g., eel and chicken calcitonin—separates itself from mammalian calcitonins by being a dual amylin and calcitonin receptor agonist (DACRA) and by possessing a markedly higher in vivo potency [14, 15]. sCT has an antiresorptive effect on the bone and chondroprotective effects in various models of osteoarthritis (OA) and RA, both ex vivo and in vivo [16]. In canine OA studies, sCT has been shown to reduce osteoarthritic lesion severity in articular cartilage [17, 18]. In ovariectomized rat models, CT treatment reduced bone turnover, prevented osteopenia [19], and decreased cartilage erosion [20]. An antiarthritic combination therapy of elcatonin (eel calcitonin derivative) and low doses of dexamethasone has been shown to strongly inhibit joint destruction markers in collagen-induced arthritis (CIA) rats [21]. Importantly, sCT had an analgesic effect in clinical studies of patients suffering from Paget’s disease, osteoporotic vertebral fractures, and other musculoskeletal disorders, although the mode of action is unknown [22–25]. An oral formulation of sCT decreased knee OA cartilage degradation in phase I and phase II clinical trials [26, 27], although it ultimately failed to meet the primary endpoint of reduction in joint space narrowing in phase III clinical trials—despite indications of improvements in WOMAC pain and biomarkers of cartilage and bone degradation [28].

KBPs (Key Bioscience peptides) are novel CT analogues with improved activity, compared to classical CTs, both at the level of potency, also in terms of prolonged receptor activation, and have been studied for their effects on obesity and diabetes [15, 29, 30]. KBP molecules have already been compared with sCT and demonstrated to have a more potent activation of the amylin and calcitonin receptors than sCT [31]. The effects on bone, cartilage, and pain are, however, not fully understood. Hence, the aim of this study was to investigate the antinociceptive effects as well as the protective effects on the bone and cartilage of a KBP administered alone or in combination with the NSAID naproxen in the CIA rat model of RA.

## Methods

### Rats

All animal procedures were performed in accordance with guidelines from the Animal Welfare Division of the Danish Ministry of Justice under the institutional license issued to Nordic Bioscience (2014-15-0201-00097). Fifty female Lewis rats aged 6–8 weeks, weighing 170–200 g, were obtained from Envigo and housed at the Nordic Bioscience animal facility (21–31 °C, 55–65% relative humidity, 12:12 h light/dark cycle, food and water ad libitum) in groups of three to four animals per cage. The rats were divided into the following groups: CIA control (*n* = 10), CIA with KBP monotherapy (CIA+KBP, *n* = 10), CIA with KBP and naproxen combination therapy (CIA+KBP+Napr, *n* = 10), CIA with naproxen monotherapy (CIA+Napr, *n* = 10), and sham (*n* = 10). The rats were randomly distributed into the groups with comparable baseline burrowing performance prior to immunization. An additional pilot experiment, with increased severity, is described in detail in Additional file 1: Supplementary Material.

### Induction of arthritis

The rats were anesthetized using isoflurane and were shaved around the base of the tail. Rats were immunized, using a protocol modified from Nielsen et al. [32], with 100 μl of 2 mg/ml porcine type II collagen (Chondrex) dissolved in 0.05 M acetic acid and emulsified 1:1 in incomplete Freund’s adjuvant injected intradermally around the base of the tail. One week later, another 100 μl of the same emulsion was injected. Rats that were not immunized were injected with saline.

### KBP and naproxen dosing regimen

The sequence of the synthetic KBP (Key Bioscience peptide) (American Peptide Company) has been published previously [33]. KBP was dissolved in saline for subcutaneous injection. The dose chosen for this study was 10 μg/kg, based on previous KBP studies in obesity [34]. Naproxen was dissolved in 1% carboxymethyl cellulose with three drops of Tween 80, all from Sigma-Aldrich (St. Louis, MO, USA), and administered at 8 mg/kg, based on previous research [35], by oral gavage. Both drugs were administered daily from day 0, the day of the first immunization.

### Health assessment and humane endpoints

After 8 days, rats were examined daily for an evaluation of disease progression. Rats were scored 0–3: 0 = normal, 1 = light disease, 2 = moderate disease, and 3 = severe disease. The parameters for the scoring were fur grooming, posture, and exploratory behavior; abnormal behaviors were scored one point each. A score of 3 was a humane endpoint, at which rats were euthanized by exsanguination under isoflurane anesthesia. After the second immunization, the rats’ health was also assessed by a joint score based on swelling of digits, as well as tarsal/carpal and metatarsal/metacarpal joints, scoring one point per swollen digit/joint. A score ≥ 10 was a humane endpoint. Frequent weight monitoring (Additional file 2: Figure S4) was also performed, and a loss of more than 20% of the rats’ baseline weight was a humane endpoint. A digital caliper was used to measure paw width, indicative of joint inflammation, three times per week.

### Mechanical allodynia

Mechanical allodynia was evaluated by measuring the withdrawal thresholds of both hind paws in response to von Frey hair filaments using the up-down method, as previously described [36]. After acclimatization to the procedure, allodynia was assessed before immunization to establish baseline and for up to 44 days post-immunization.

### Cold hypersensitivity

A drop of acetone was applied to the hind paws from below, and the reactions were recorded and scored using a system based on previous publications [37, 38]. If animals did not react within 20 s of acetone application, they were scored 0. If they responded within 20 s, animals were monitored for an additional 20 s. The rats were scored 0–4: 0 = no response; 1 = quick withdrawal, flick, or stamp of the paw (total reaction time < 1 s); 2 = prolonged withdrawal or repeated flicking (total reaction time 1–3 s); 3 = repeated flicking together with licking at the ventral side of the paw (total reaction time 3–10 s); and 4 = prolonged licking (total reaction time > 10 s). The test was performed three times per paw with minimum 10 min in between, and the scores were added, resulting in a maximum possible score of 24. Cold hypersensitivity was measured at baseline and 2 and 3 weeks post-immunization.

### Burrowing test

The burrowing test, as previously described [39], was used to test for analgesic effects of the treatments. Plastic tubes (32 cm long × 10 cm diameter) were capped at one end and angled so that the open end was 60 mm above the cage floor. The tubes were filled with 2.5 kg pea shingle gravel (Lavpris Dyrehandel). After acclimatization to the test, rats were left in empty cages with a paper on the floor for 30 min and subsequently allowed to interact with the burrowing tube for 60 min followed by weighing of the gravel remaining in the tubes to calculate the weight of the displaced gravel. The rats were randomized into treatment groups with comparable baseline burrowing performance. The burrowing test was performed once per week.

### Biochemical analysis

Blood samples (0.5 ml) were collected from the caudal vein 2 h post-dosing, after overnight fasting, prior to immunization, and at day 29. At the study termination (day 44), the rats were euthanized and blood was collected directly from the jugular vein. All biomarkers were measured in plasma. C3M, an enzyme-linked immunosorbent assay (ELISA) specific for mature type III collagen degraded by MMP-9, was measured as previously described [40]. CTX-I, a biomarker of bone degradation detecting C-terminal telopeptides of type I collagen, was measured using RatLaps (CTX-I) EIA (IDS) according to the manufacturer’s instructions.

### Histology

The right hind paws were collected from the animals and were fixed in 10% formalin for a week, followed by decalcification in 15% EDTA. The decalcified paws were divided sagittally between metatarsals II and III, in order to expose the lateral tibiotarsal region, using a scalpel. The paws were infiltrated with paraffin using a Tissue-Tek VIP 5 Jr. (Sakura Finetek), embedded in paraffin, and cut into 5-μm-thick sagittal sections using a HM 360 microtome (Microm International GmbH). The sections were deparaffinized according to standard procedures prior to stainings.

Safranin O (SafO) + Fast green staining (*n* = 10/group) was used to assess cartilage degradation. Tartrate-resistant acid phosphatase (TRAP; *n* = 4/group) staining, counterstained with Mayer’s hematoxylin, was performed as previously described [41] to detect osteoclasts. For the immunohistochemistry (*n* = 4/group), antigen retrieval was performed overnight in sodium citrate buffer (10 mM sodium citrate, 0.05% Tween 20, pH 6.0) at 60 °C. Endogenous peroxidase activity was blocked by incubating with 1.2% hydrogen peroxide in 70% ethanol (30 min), followed by blocking in a solution of 0.5% casein in tris-buffered saline with 0.1% Tween 20 and 1% Triton X-100 (TBS-T, 20 min) to reduce nonspecific antibody binding. Primary antibodies against CD68 (Abcam, cat. ab125212, 1:1500), p75 (Abcam, cat. ab8874, 1:4000), and transient receptor potential cation channel subfamily V member 1 (TRPV1; Abcam, cat. ab31895, 1:2500) were diluted in the blocking solution and incubated overnight at 4 °C. Super Sensitive Polymer-HRP IHC Detection System (BioGenex, cat. QD420-YIKE) was used, according to the manufacturer’s instructions, to develop the stainings. All steps were performed at room temperature, unless otherwise specified, and all sections were washed with TBS-T between steps. Finally, the sections were counterstained with Mayer’s hematoxylin.

After the stainings, the sections were dehydrated according to standard procedures and mounted in Pertex. Digital micrographs were obtained with a DP71 camera connected to a BX60 microscope with × 4 and × 10 objectives using the Olympus cellSens software (Olympus) and were used for qualitative analysis.

### μCT analysis

The left hind paws of all rats were removed and fixed in 4% paraformaldehyde for a day and were thereafter stored in 70% ethanol at room temperature. In order to quantify the bone structure, the paws were scanned with a high-resolution microcomputed tomographic (μCT) system (vivaCT 40, Brüttisellen, Scanco Medical AG) set to 70 kVp and 114 μA. The two regions of interest (ROI) were the metatarsophalangeal joints of the three middle toes and the tibiotarsal joint, as indicated in Fig. 5. Each scan took approximately 30 min and created 420 μCT slide images (image projections), of which 365 slide images (image projections) (3832.5 μm) covering the entire joint and joint space were used for the analysis of the bone microstructure. All μCT images resulted in 3D reconstruction cubic voxel sizes of 10.5 × 10.5 × 10.5 μm (2048 × 2048 × 2048 pixels) with 32-bit gray levels. The images were then segmented to obtain accurate 3D imaging datasets, as previously described [42]. Due to the irregular structure of metatarsophalangeal and tibiotarsal joints, the majority of the bone tissue of the region of interest was dominated by the cortical bone with a portion of the trabecular bone (Fig. 5). Contouring was made including the cortical bone and partial trabecular bone (green line) (Fig. 5b, c). This line was along the external surface of the ROI, including both cortical bone and trabecular bone, and minimizing joint space. Bone volume to total tissue volume fraction (BV/TV) and trabecular thickness (Tb.Th) were calculated based on assumption-free 3D methods.

### Statistical analyses

All data, except μCT data, were statistically analyzed by repeated measures two-way analysis of variance (ANOVA) with Tukey’s multiple comparisons test against all groups, with the assumption that the data were normally distributed. The μCT data were analyzed by one-way ANOVA with Tukey’s multiple comparisons test against all groups, with the assumption that the data were normally distributed. Acetone test and burrowing data were normalized by subtracting baseline (BL) values from other values for each rat; von Frey data were normalized as percent change from baseline; biomarker data were normalized as x-fold of baseline. The last observation carried forward was used for all euthanized rats. All data are presented as mean ± standard error of the mean (SEM). Statistical significance was considered to be *P* values < 0.05. All plots were generated in GraphPad Prism 7.01 (Graph Pad Inc).

## Results

### Naproxen, but not KBP, delays humane endpoint following CIA induction

**Fig. 1:**
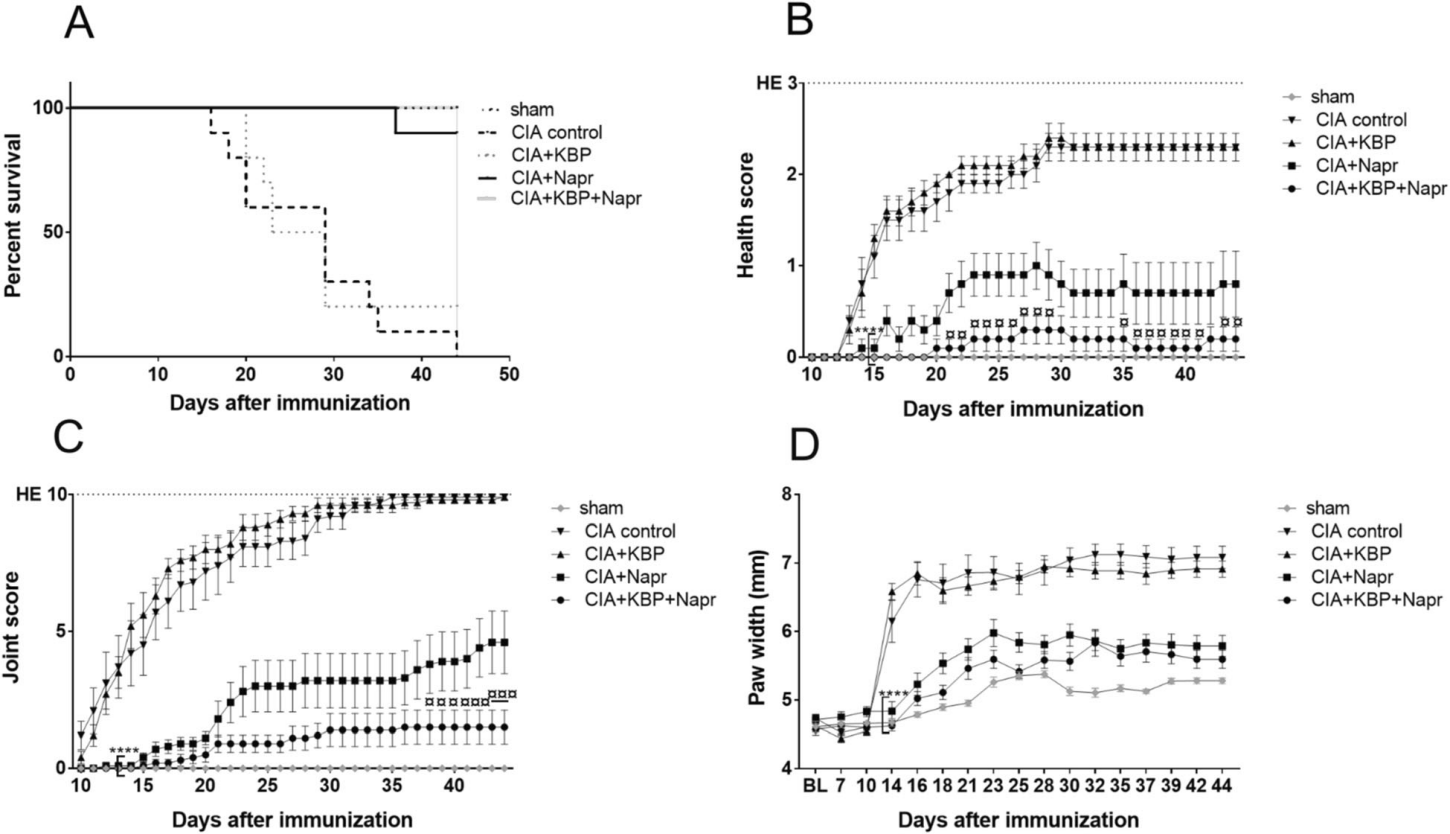
Effects of KBP and naproxen on health and inflammation status. The time until any humane endpoint is presented as a Kaplan-Meier curve (**a**). Health and inflammation status was assessed using behavioral health scores (**b**), counting of swollen joints (**c**), and paw width measurements (**d**). Data in **b**–**d** are presented as the mean ± SEM, *n* = 10/group, using the last observation carried forward for euthanized rats. Asterisk (*) indicates statistical comparisons to CIA control, and currency sign (¤) indicates comparisons of CIA+Napr and CIA+KBP+Napr. *^/¤^*P* < 0.05; **^/¤¤^*P* < 0.01; ***^/¤¤¤^*P* < 0.001; ****^/¤¤¤¤^*P* < 0.0001

To assess whether KBP monotherapy or in combination with naproxen could decrease disease activity in CIA rats, the time until the rats reached any humane endpoint was investigated. Of the 50 rats, 18 (36%) reached a humane endpoint. The time till termination of the CIA control group was 29 days, with only 10% remaining at termination (Fig. 1a). With KBP monotherapy, mean survival was 26 days with 20% alive at termination. Naproxen monotherapy or in combination with KBP resulted in 90% and 100%, respectively, of rats reaching the termination of the experiment. However, the strong effect of naproxen monotherapy prevented investigation into synergistic effects of the combination therapy on time till humane endpoint. We observed such a combination effect in a pilot experiment using a more aggressive model (Additional file 2: Figure S1A), indicating that the combination therapy may be beneficial but that it is not detectable in the more benign model due to the potency of naproxen.

### KBP and naproxen combined improves health scores

The behavioral health score of CIA control rats compared to that of sham rats worsened from day 14 (*P* = 0.0024) until termination (Fig. 1b). KBP did not improve the health score, whereas naproxen monotherapy resulted in a significant improvement from day 14 onward (*P* = 0.0119). The combination therapy further improved health scores relative to the CIA control from day 14 onward (*P* = 0.0024, day 14 onward), as well as compared to naproxen monotherapy from day 21 onward (*P* = 0.0476). Similar results were seen in the pilot study (Additional file 2: Figure S1B).

### KBP and naproxen combined reduces joint swelling

The CIA control group was the first to demonstrate evidence of clinical inflammation as measured by joint score (*P* = 0.0007, day 12) and paw width (*P* < 0.0001, day 14) compared to the sham group (Fig. 1c, d). KBP monotherapy had no effect on either score. Paw width of the naproxen and the combination therapy groups were significantly improved from day 14 (*P* < 0.0001). The joint score in the combination group was significantly improved compared to naproxen monotherapy (day 38, *P* = 0.0272; day 44, *P* = 0.0007), with a similar trend in paw width. In the aggressive model, the combination therapy was significantly improved over naproxen monotherapy in both scores (Additional file 2: Figure S1 C-D).

### KBP and naproxen combined reduces mechanical allodynia and cold hypersensitivity

**Fig. 2:**
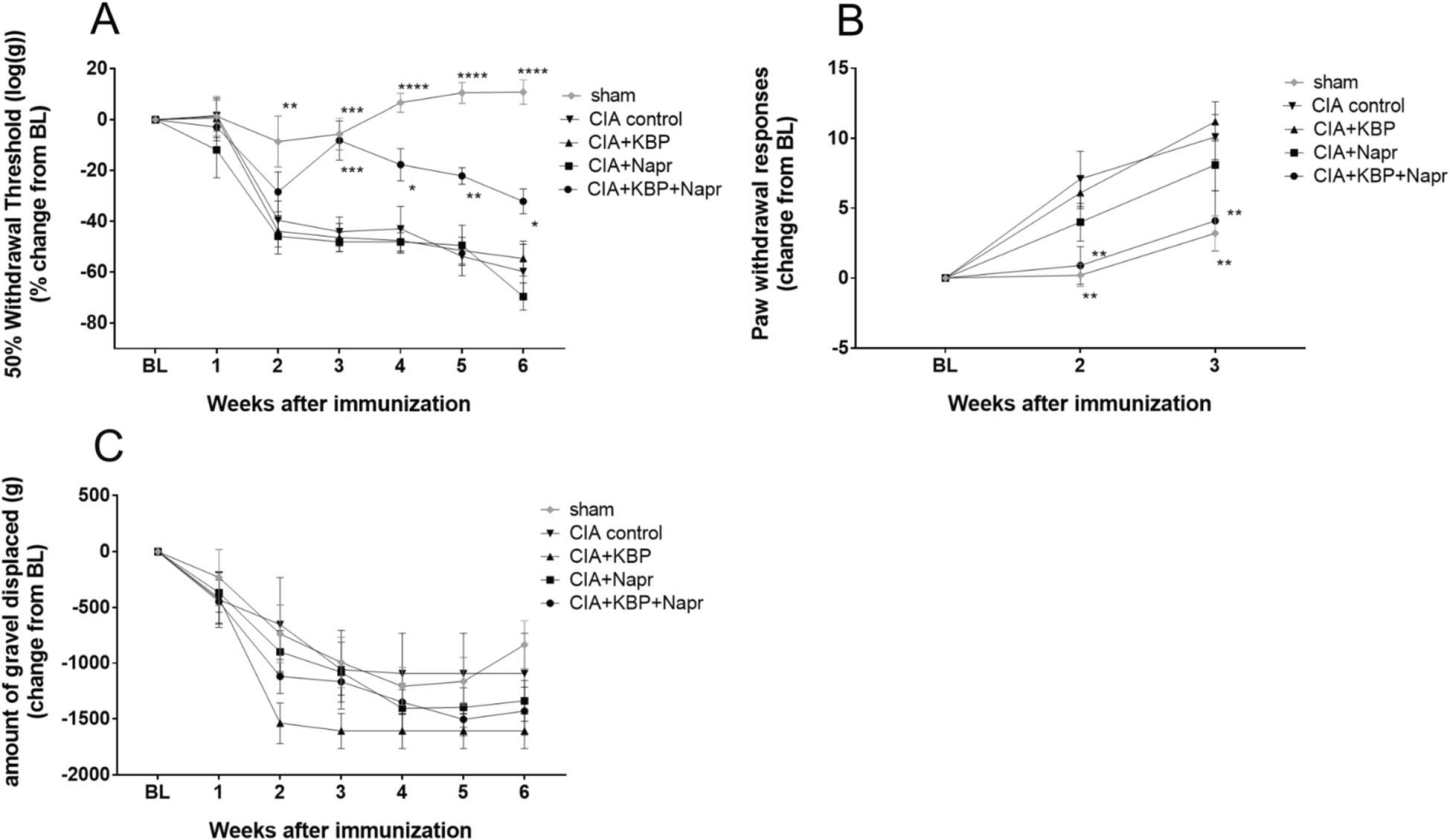
Analgesic effects of KBP and naproxen. Analgesic effects on mechanical allodynia measured with von Frey filaments and the up-down method (**a**), on cold hypersensitivity measured with the acetone test (**b**), and on innate burrowing behavior measured with the burrowing test (**c**). Data in **a** are presented as the percent change from baseline, and data in **b** and **c** are presented as the mean change from baseline, all ± SEM, *n* = 10/group, using the last observation carried forward for euthanized rats. Asterisk (*) indicates statistical comparisons to CIA control.*P < 0.05; **P < 0.01; ***P < 0.001; ****P < 0.0001

To characterize the effects of the KBP and naproxen on pain sensitization, we analyzed the mechanical allodynia, cold hypersensitivity, and burrowing behavior in the rats. Mechanical allodynia (Fig. 2a) was observed in the CIA control from week 2 onward (*P* = 0.0046). KBP or naproxen monotherapy showed no effect on the allodynic response. However, the combination therapy led to a significant reduction in allodynia from week 3 onward (*P* = 0.0006, compared to CIA control). Due to the severity of the more aggressive model, mechanical allodynia could not be measured in the pilot study.

Cold hypersensitivity was observed at both time points (Fig. 2b, *P* = 0.0016) in the CIA control group. KBP or naproxen monotherapy had no effects. However, the combination of naproxen and KBP therapy greatly reduced the cold hypersensitivity at 2 and 3 weeks after immunization, compared to the CIA group (*P* = 0.0058; *P* = 0.0083). No effects could be detected for any compound, or even CIA versus sham, in the pilot study (Additional file 2: Figure S2A), likely due to the highly aggressive model and sedentary behavior of the rats.

As for the burrowing model, at baseline, all rats showed the same vigorous burrowing behavior but the burrowing decreased over time in all groups, including sham (Fig. 2c). KBP and naproxen, alone or combined, did not show any significant increase at any time point, although these data should be interpreted cautiously given the low sensitivity of the test and overall decrease in burrowing even in the sham group. Similar trends were seen in the pilot study (Additional file 2: Figure S2B).

### KBP suppresses bone resorption but neither KBP nor naproxen reduces type III collagen degradation

**Fig. 3:**
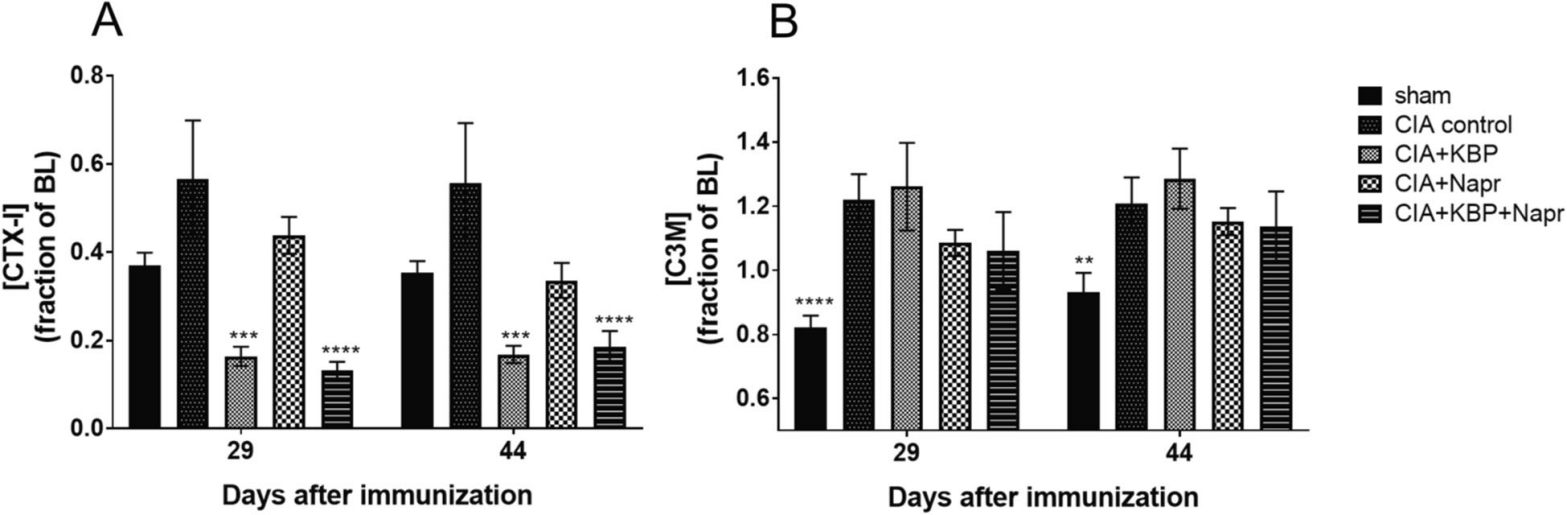
Effects of KBP and naproxen on bone resorption and type III collagen degradation. Bone resorption was assessed by CTX-I (**a**) and type III collagen degradation by MMP-9 was assessed using C3M (**b**). CIA control *n* = 6; CIA+KBP *n* = 5; CIA+KBP+Napr *n* = 10; CIA+Napr *n* = 10; sham *n* = 10. Data are presented as the mean fraction of each rat’s baseline measurement ± SEM using the last observation carried forward for euthanized rats. Asterisk (*) indicates statistical comparisons to CIA control.*P < 0.05; **P < 0.01; ***P < 0.001; ****P < 0.0001

To characterize the effect of KBP on bone turnover, CTX-I (Fig. 3a) was measured in plasma. KBP monotherapy or combined with naproxen suppressed bone resorption at day 29 (*P* = 0.0005; *P* < 0.0001) and day 44 (*P* = 0.0008; *P* = 0.0001). Naproxen therapy did not result in any significant effects.

C3M is a marker of synovial inflammation, generated by the cleavage of type III collagen by MMPs during inflammation [43]. C3M (Fig. 3b) was increased in plasma in the CIA control group 29 and 44 days post-immunization (*P* < 0.0001; *P* = 0.0029). Monotherapy with KBP or naproxen did not show any significant effect on C3M, but there was a trend towards decrease C3M levels in the naproxen and the combination groups. However, in the aggressive model system (Additional file 2: Figure S3), the combination therapy was significantly different when compared to the CIA control at days 28 and 39 (*P* = 0.0069; *P* = 0.0078).

### KBP and naproxen combined reduces bone and cartilage structural damage

**Fig. 4:**
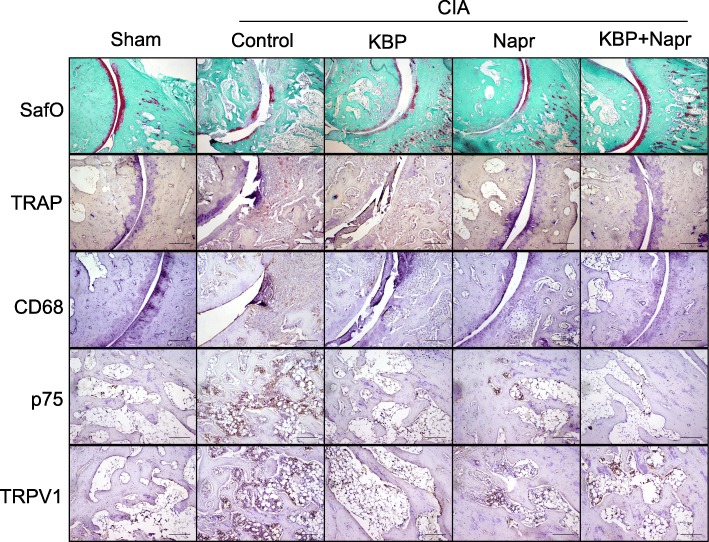
KBP and naproxen effects on ankle joint structure and immunohistochemical markers. Tibiotarsal joints were stained using the following procedures: SafO + Fast green staining to assess cartilage damage, TRAP and Mayer’s hematoxylin staining for the detection of osteoclasts, and CD68 and Mayer’s hematoxylin staining for the detection of macrophages. The bone marrow was stained using the following procedures: p75 and Mayer’s hematoxylin staining and TRPV1 and Mayer’s hematoxylin staining, both to assess innervation. Representative micrographs are shown for *n* = 10/group for the SafO staining and *n* = 4/group for the other stainings. Scale bars indicate 200 μm

Sections were stained with SafO + Fast green (Fig. 4) to assess cartilage damage. CIA induction led to extensive cartilage degradation, which was not observed in the sham animals. Naproxen monotherapy appeared to reduce joint degradation and preserve chondrocyte organization, consistent with other studies [44], while KBP monotherapy had little to no effect. The combined therapy resulted in the normalization of the joint phenotype by greatly reducing joint destruction and chondrocyte rearrangement.

In order to visualize the osteoclast distribution, sections were stained for TRAP (Fig. 4). Large parts of the joint in the CIA control and KBP monotherapy groups showed high numbers of TRAP-positive cells. Fewer TRAP-positive cells were seen in the naproxen and combination therapy groups, supporting other studies reporting that NSAIDs inhibit osteoclastogenesis in vitro [45].

### KBP and naproxen effects on markers of inflammation and pain

Macrophage infiltration was assessed by CD68 staining (Fig. 4). The CIA control group stained highly positive for CD68, indicating ongoing inflammation, with little to no staining in the sham group. In contrast, CD68 staining was less pronounced in both the naproxen and KBP monotherapy groups. The combination therapy resulted in fewer CD68-positive cells which is consistent with the joint score and paw width data.

The p75 (low-affinity nerve growth factor receptor) staining, previously shown to correlate with inflammation [46], suggests a high level of innervation of the bone marrow in the CIA control rats, which appeared decreased by both naproxen or KBP monotherapy (Fig. 4). The combination therapy resulted in even less p75 staining in the bone marrow, which could be related to the analgesic effects found in the pain outputs.

TRPV1, a nonselective calcium channel, is mostly found in nociceptive neurons of the peripheral nervous system, and TRPV1 staining represents inflammation and pain [47]. Increased TRPV1 staining was seen in the CIA control rats, indicating increased pain and increased inflammation (Fig. 4). Slightly less staining was observed in both therapies alone or combined, indicating an effect associated with pain propagation.

### KBP and naproxen combined reduces bone microstructural damage

**Fig. 5:**
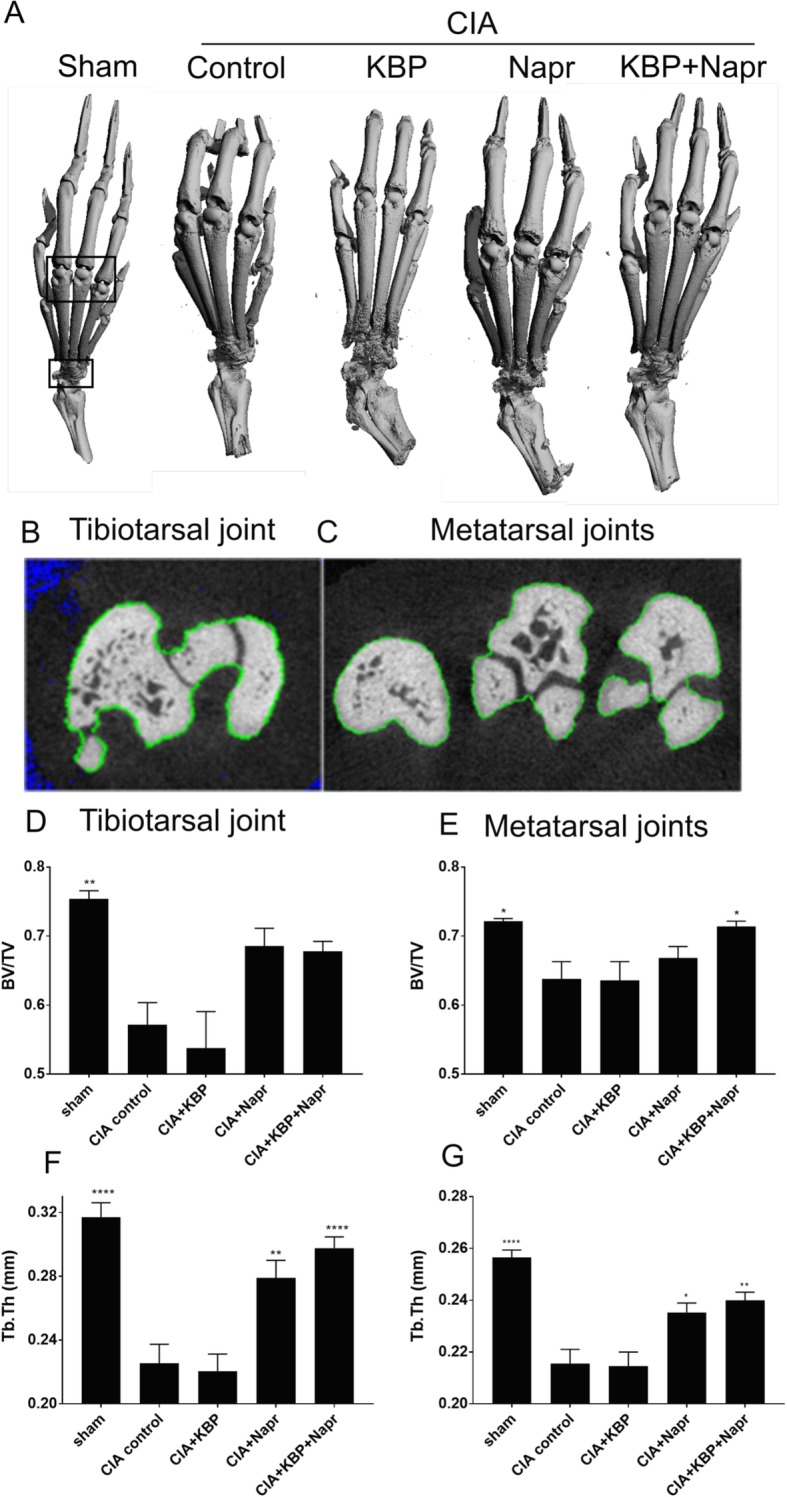
KBP and naproxen effects on bone microstructure. μCT was performed to analyze structural effects of CIA and the treatments. Representative 3D reconstructions (**a**) with regions of interest (black boxes) and 2D contouring (**b**, **c**) indicate the area for three tibiotarsal (**d**, **f**) and metatarsal (**e**, **g**) analyses. The exact of bone volume to total volume (BV/TV, **d**, **e**) and trabecular thickness (Tb.Th, **f**, **g**) are reported. The data are presented as the means ± SEM of *n* = 10/group. Asterisk (*) indicates statistical comparisons to CIA control. *P < 0.05; **P < 0.01; ***P < 0.001; ****P < 0.0001

CIA resulted in a significant reduction in the tibiotarsal and metatarsophalangeal joints BV/TV (Fig. 5d, e) and Tb.Th (Fig. 5f, g). Naproxen therapy did not significantly alter the tibiotarsal (*P* = 0.1025, Fig. 5d) or metatarsophalangeal BV/TV (*P* = 0.7841, Fig. 5e), while a significant effect was observed for the combination therapy in the metatarsophalangeal joints (*P* = 0.0427), but not in the tibiotarsal joint (*P* = 0.1278). Naproxen therapy significantly improved tibiotarsal (*P* = 0.0053, Fig. 5f) and metatarsophalangeal Tb.Th (*P* = 0.0215, Fig. 5g), and the combination therapy further increased these effects (*P* < 0.0001; *P* = 0.0019).

## Discussion

There is an unmet need for safer and more efficacious RA therapies that can effectively halt disease progression and preserve the joint structure [48]. In this study, we demonstrate that combining KBP and naproxen is a valid therapeutic strategy for reducing joint destruction and pain in chronic inflammatory arthritis. More specifically, our study shows that combining a KBP with an NSAID (1) improves overall health, (2) reduces pain, (3) reduces inflammation, and (4) protects the bone and cartilage through a combination of anti-inflammatory and structural effects. Our hypothesis is that DACRAs, due to their high potency [15] and ability to reduce bone and cartilage turnover [17], in combination with an anti-inflammatory treatment can provide clinical benefits in RA by reducing the destructive inflammation and thereby allow the potential bone- and cartilage-protective effects of the KBP to manifest, resulting in an improved outcome.

In this study, we demonstrate that combining naproxen with a KBP added significant improvement over naproxen monotherapy. KBP monotherapy did not have significant effects in most structural parameters, similarly to what has previously been reported for CT monotherapy in the CIA model [21, 49]. This may in part be due to the severity of the disease induction being too aggressive for the KBP to overcome, which can be seen within the time to humane endpoint data. Combination therapy resulted in maintained bone structure and absence of cartilage lesions, similarly to previous sCT and CT monotherapy OA studies [17, 50]. A previous study combining CT with glucocorticoids, instead of NSAIDs, also demonstrated a chondroprotective effect in the CIA model [21], indicating a beneficial effect of inhibiting bone destruction and inflammation on joint integrity in severe inflammatory arthritic conditions.

KBP monotherapy had a limited effect on pain, as assessed in our study, but in combination with naproxen, we found a significant improvement in two pain outputs. Reductions in pain have previously been reported for sCT, both clinically [27] and pre-clinically [51], but not in relation to rheumatoid arthritis. The lack of efficacy of KBP monotherapy on pain could be due to the severity or the substantial inflammation in the model; potentially, KBPs may be able to alleviate pain on their own in arthritic diseases with less inflammation, as has been shown with CT [50]. Further studies, e.g., in less inflamed arthritis models, are needed to fully understand the effects and mode of action of KBP on joint structure and pain.

In our study, naproxen therapy was crucial for the improvement of overall health and inflammation scores. Interestingly, we observed trends indicating a potential synergistic effect when combining naproxen with KBP in these parameters, but a broader naproxen dose range is needed to validate these effects. A reliable biomarker which might be relevant for the mechanism of action of the combination therapy might be CXCL5 as shown in the CIA study using eCT and dexamethasone [21]. However, an equally important observation could be the elevation of C3M fount in the CIA rats which was subsequently reduced by treatment with the combination therapy. Several studies have linked C3M with treatment responses within RA patients [52, 53] as well as with joint replacement in patients with OA [54]. The clinical scoring data and paw width data align with the reduction in CD68 staining [55] and decrease of C3M, as it emphasizes the importance of naproxen in halting inflammation in order to prevent joint damage. Moreover, high levels of inflammation are associated with increased number of osteoclasts [56], and here, we found that the TRAP-positive cells were profoundly reduced with naproxen and combination therapy.

We speculate that combination therapy have an improved effect due to the drugs acting independently on two different aspects of the disease: naproxen targets the inflammatory driver while KBP acts on the bone and cartilage structure in addition to potential intrinsic analgesic effects, thereby reducing three disease processes that may contribute to RA pain. The antiresorptive properties of KBP, and potentially sCT at different doses, may reduce the bone and cartilage degradation in RA animal models, when the cascade of cytokine-mediated inflammatory stimulus is suppressed using an NSAID.

As this is the first time we have tested KBP together with an NSAID within a RA animal model, future studies should also test and compare sCT with NSAIDs in vivo as well as in vitro, in articular cartilage explants. Furthermore, the aggressiveness of the model, as previously discussed, may hide some of the effects when using either drug alone. The CIA RA model has been widely employed in the assessment of pharmacological treatment efficacy [57, 58]. We found that the rats’ hind limbs became severely inflamed 2 weeks post-immunization, which may indicate a less severe phenotype in our study compared with other studies [59] and the more aggressive pilot study. However, the consistent data across two different severities of the model support the relevance of the output. Due to the predefined humane endpoints, animals had to be terminated at different time points of the study, which limited the statistical interpretation but still gave a very important biological output. Additionally, only a subset of animals were used for qualitative, rather than quantitative, histological analyses—hence, the histology requires cautious interpretation.

Notwithstanding the limitations, this study has several strengths. Several pain evaluation techniques and joint structure analyses were employed to cross-validate both the potential analgesic effects of the drugs and to characterize joint structure damage. Importantly, across two studies using models of different severity, we found the same output, namely that KBP in combination with naproxen improves both the clinical outcome and pain scores.

## Conclusions

The current study indicates that low doses of an NSAID combined with a dual amylin calcitonin receptor agonist, as KBP, has significant therapeutic effects on joint structure and pain in an animal model of severe inflammatory arthritis and may allow for improved clinical outcomes with reduced side effects.

## Additional files

Additional file 1: The combination effect of a dual amylin and calcitonin receptor agonist and naproxen in a more severe collagen-induced arthritis model. (DOCX 26 kb)

Additional file 2: **Figure S1.** Pilot study effects of KBP and naproxen on health and inflammation status. **Figure S2.** Pilot study analgesic effects of KBP and naproxen.. **Figure S3.** Pilot study effects of KBP and naproxen on type III collagen degradation. **Figure S4.** Body weight monitoring. (ZIP 197 kb)

## Abbreviations

C3M: MMP-9 proteolytically revealed neo-epitope of type III collagen
CIA: Collagen-induced arthritis
CT: Calcitonin
CTX-I: C-terminal telopeptides of collagen type I
DACRA: Dual amylin calcitonin receptor agonist
DMARDs: Disease-modifying antirheumatic drugs
EDTA: Ethylenediaminetetraacetic acid
HCl: Hydrogen chloride
IL-6: Interleukin 6
MMP-9: Matrix metallopeptidase 9
NSAIDs: Nonsteroidal anti-inflammatory drugs
OA: Osteoarthritis
RA: Rheumatoid arthritis
RT: Room temperature
sCT: Salmon calcitonin
TBS: Tris-buffered saline
TNF: Tumor necrosis factor
TRAP: Tartrate-resistant acid phosphate
WOMAC: Western Ontario and McMaster Universities Arthritis Index
μCT: Microcomputed tomography
