# Supplementary material for: Combining naproxen and a dual amylin and calcitonin receptor agonist improves pain and structural outcomes in the collagen-induced arthritis rat model
Source: Arthritis Res Ther. 2019 Feb 22;21:68. doi: 10.1186/s13075-019-1819-9 (PMC6387482; doi:10.1186/s13075-019-1819-9)
Supplement: Supplementary file 1 — The combination effect of a dual amylin and calcitonin receptor agonist and naproxen in a more severe collagen-induced arthritis model. (DOCX 26 kb) [file 13075_2019_1819_MOESM1_ESM.docx]

**Additional file 1**

# Methods

This pilot study was performed as described for the main study, except for the following changes. Forty-seven female Lewis rats aged 8–10 weeks, weighing 180–220 g at the start of the study, were used in the pilot. The treatment groups were: CIA control (n=10); CIA with KBP monotherapy (n=10); CIA with KBP and naproxen combination therapy (n=10); CIA with naproxen monotherapy (n=9); sham (n=8). Rats were immunized with a higher dose, 450 μl, of the 2 mg/ml porcine type II collagen emulsion, as previously described [1]. A 450 µl booster injection of the same emulsion was given one week after the first injection. Treatment regimen were initiated two days after the booster injection, rather than at the first injection as in the main study. Due to the severity of the more aggressive model, mechanical allodynia could not be measured.

# Results

## KBP and naproxen delays humane endpoint following CIA induction.

The median time till human endpoint of the CIA control group was 21.5 days (Additional file 1. Figure 1A) after the first immunization, indicating increased severity of disease compared to the main study. The KBP group mean time till human endpoint was 20.5 days, while the naproxen therapy improved the time till human endpoint, resulting in 22% remaining at termination and 80% remaining after combination therapy.

## KBP and naproxen combined improves health scores.

The CIA control rats had a worsening behavioral health score from day 16 (P=0.0151) until their termination (Additional file 1. Figure 1B). KBP monotherapy did not improve the health score, but naproxen monotherapy and combination therapy resulted in an improved score already after 17 days (P=0.0006; P<0.0001), and the combination therapy improved the health score more than naproxen monotherapy from day 41 onward (P=0.0109).

## KBP and naproxen combined reduces joint swelling.

Inflammation, in the form swollen joints as measured by joint score (Additional file 1. Figure 1C) and paw width (Additional file 1. Figure 1D), was detected after 15 days in the CIA control and KBP groups. KBP did not result in any improvement. Joint score deterioration had a delayed onset with naproxen monotherapy and the combination therapy slowed it further; after 27 days the combination was a significant improvement over naproxen alone (P=0.0491). Similarly, combination therapy had significantly improved paw width over naproxen alone after 36 days (P=0.0157).

## The treatments had no effect on cold hypersensitivity and burrowing performance.

Cold hypersensitivity was not found to be significantly different between the sham and CIA control animals, and the different treatments did not reduce the hypersensitivity (Additional file 1. Figure 2A).

In the burrowing test, all rats showed the same vigorous burrowing behavior at baseline (Additional file 1. Figure 2B). All the immunized rats had decreased burrowing performance over time. After one week, naproxen (P<0.0006) and combination groups (P<0.0001) burrowed significantly more than the CIA control group. KBP monotherapy did not result in any significant effects, and from two weeks onward there was no significant effect of any compounds.

## KBP and naproxen combined reduces type III collagen degradation.

C3M was increased in the CIA control group when compared to the sham animals (Additional file 1. Figure 3, P<0.0001 for all time points). Combination therapy significantly reduced C3M after both 28 and 39 days (P=0.0069; P=0.0078), but neither compound alone had significant effects.

## Body weight monitoring as indicator of disease severity.

Body weight measurements were obtained frequently throughout the study in order to monitor disease activity and health status of animals (Additional file 1. Figure 4). Body weight loss of 20% of their original weight was a humane endpoint. All the immunized animals lost weight, particularly in the more severe pilot study (Additional file 1. Figure 4A). KBP is known to result in weight loss [2] and therefore the rats were expected to lose weight, and CIA+KBP rats lost even more weight than the CIA control. The combination therapy and naproxen alone resulted in more normalized weight, particularly in the main study (Additional file 1. Figure 4B), but they still weighed less than the sham group which increased in weight due to normal growth.

**References**

1. Siebuhr AS, Wang J, Karsdal M, Bay-Jensen A-C, Y J, Q Z. Matrix metalloproteinase-dependent turnover of cartilage, synovial membrane, and connective tissue is elevated in rats with collagen induced arthritis. J Transl Med [Internet]. 2012;10:195. Available from: http://www.ncbi.nlm.nih.gov/pubmed/22992383

2. Gydesen S, Hjuler ST, Freving Z, Andreassen KV, Sonne N, Hellgren LI, et al. A novel dual amylin and calcitonin receptor agonist, KBP-089, induces weight loss through a reduction in fat, but not lean mass, while improving food preference. Br J Pharmacol. 2017;174:591–602.

# Figure legends

**Additional file 1. Figure 1: Pilot study effects of KBP and naproxen on health and inflammation status.** The time until any humane endpoint is presented as a Kaplan-Meier curve (A). Health and inflammation status was assessed using behavioral health scores (B), counting of swollen joints (C), and paw width measurements (D). CIA control (n=10); CIA+KBP (n=10); CIA+KBP+Napr (n=10); CIA+Napr (n=9); sham (n=8). Data in B–D are presented as the mean ± SEM, using last observation carried forward for euthanized rats. * indicates statistical comparisons to CIA control and ^¤^ indicates comparisons of CIA+Napr and CIA+KBP+Napr. *^/¤^P<0.05; **^/¤¤^P<0.01; ***^/¤¤¤^P<0.001; ****^/¤¤¤¤^P<0.0001.

**Additional file 1. Figure 2: Pilot study analgesic effects of KBP and naproxen.** Analgesic effects on cold hypersensitivity measured with the acetone test 14 days after first drug dosing (A), and innate burrowing behavior (B). CIA control (n=10); CIA+KBP (n=10); CIA+KBP+Napr (n=10); CIA+Napr (n=9); sham (n=8). Data are presented as the mean change from baseline, all ± SEM, using last observation carried forward for euthanized rats. * indicates statistical comparisons to CIA control.*P<0.05; **P<0.01; ***P<0.001; ****P<0.0001.

**Additional file 1. Figure 3: Pilot study effects of KBP and naproxen on type III collagen degradation.** Type III collagen degradation by MMP-9 was assessed using C3M. CIA control n=6; CIA+KBP n=9; CIA+KBP+Napr n=10; CIA+Napr n=10; sham n=8. Data are presented as the mean fraction of each rat’s baseline measurement ± SEM using last observation carried forward for euthanized rats. * indicates statistical comparisons to CIA control and ^¤^ indicates comparisons of CIA+Napr and CIA+KBP+Napr. *^/¤^P<0.05; **^/¤¤^P<0.01; ***^/¤¤¤^P<0.001; ****^/¤¤¤¤^P<0.0001.

**Additional file 1. Figure 4: Body weight monitoring.** Weight change expressed as a fraction of baseline in the pilot study (A) and the main study (B). Data are presented as mean fraction of individual baseline, ± SEM, using last observation carried forward for euthanized rats.
