# Supplementary figures and images for: Combining naproxen and a dual amylin and calcitonin receptor agonist improves pain and structural outcomes in the collagen-induced arthritis rat model
Source: Arthritis Res Ther. 2019 Feb 22;21:68. doi: 10.1186/s13075-019-1819-9 (PMC6387482; doi:10.1186/s13075-019-1819-9)

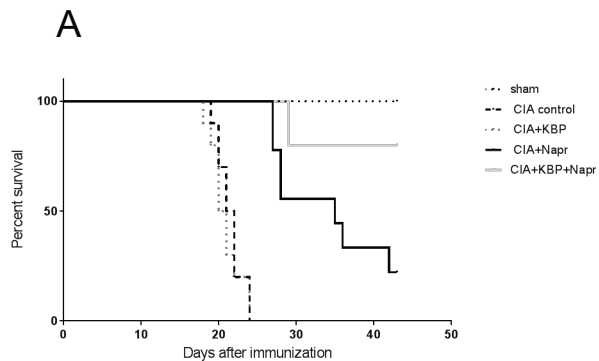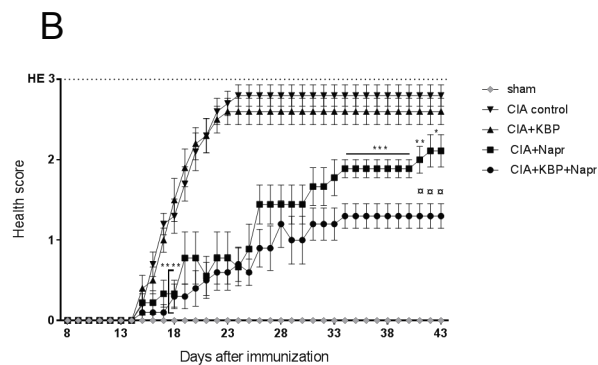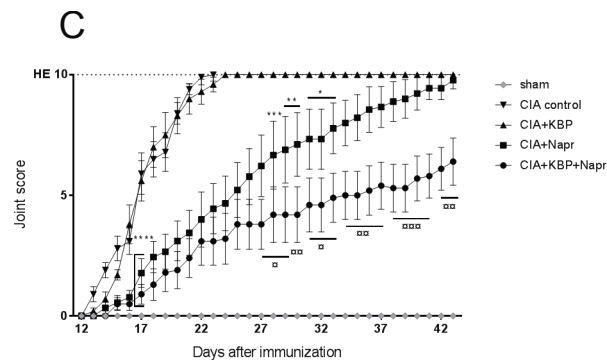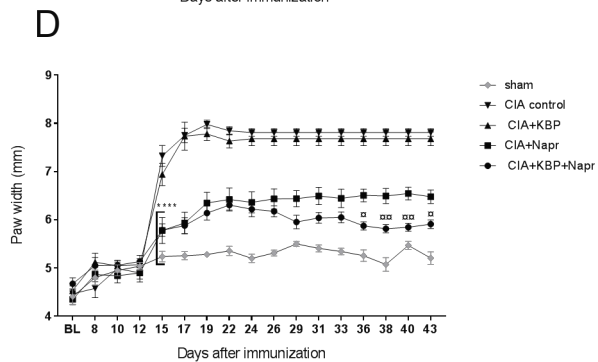

Supplement: Supplementary file 2 — Figure S1. Pilot study effects of KBP and naproxen on health and inflammation status. Figure S2. Pilot study analgesic effects of KBP and naproxen.. Figure S3. Pilot study effects of KBP and naproxen on type III collagen degradation. Figure S4. Body weight monitoring. (ZIP 197 kb) [file 13075_2019_1819_MOESM2_ESM.zip › Additional file 1. Figure 1.pdf]

A

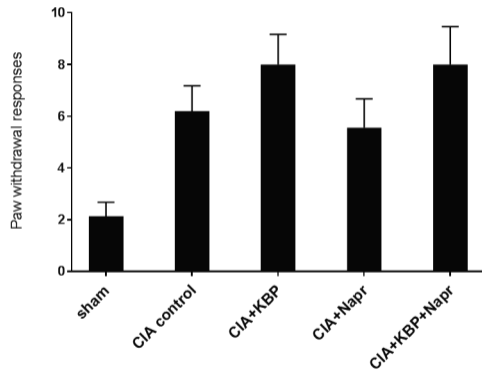

B

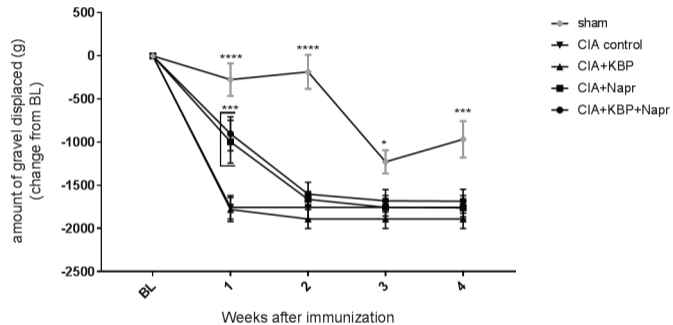

Supplement: Supplementary file 2 — Figure S1. Pilot study effects of KBP and naproxen on health and inflammation status. Figure S2. Pilot study analgesic effects of KBP and naproxen.. Figure S3. Pilot study effects of KBP and naproxen on type III collagen degradation. Figure S4. Body weight monitoring. (ZIP 197 kb) [file 13075_2019_1819_MOESM2_ESM.zip › Additional file 1. Figure 2.pdf]

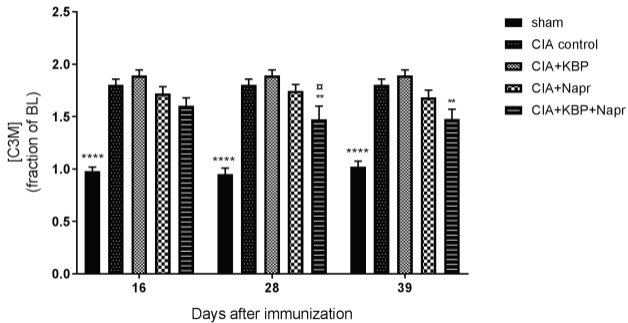

Supplement: Supplementary file 2 — Figure S1. Pilot study effects of KBP and naproxen on health and inflammation status. Figure S2. Pilot study analgesic effects of KBP and naproxen.. Figure S3. Pilot study effects of KBP and naproxen on type III collagen degradation. Figure S4. Body weight monitoring. (ZIP 197 kb) [file 13075_2019_1819_MOESM2_ESM.zip › Additional file 1. Figure 3.pdf]

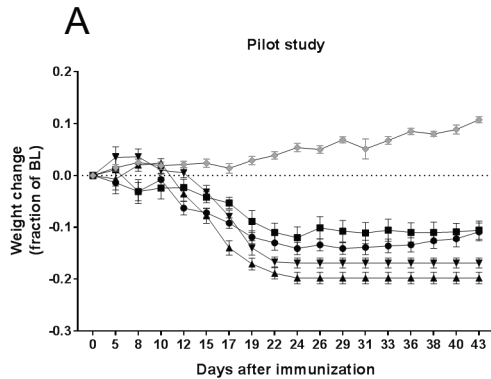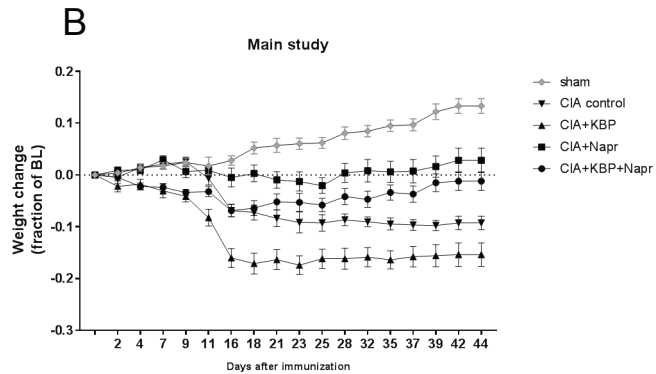

Supplement: Supplementary file 2 — Figure S1. Pilot study effects of KBP and naproxen on health and inflammation status. Figure S2. Pilot study analgesic effects of KBP and naproxen.. Figure S3. Pilot study effects of KBP and naproxen on type III collagen degradation. Figure S4. Body weight monitoring. (ZIP 197 kb) [file 13075_2019_1819_MOESM2_ESM.zip › Additional file 1. Figure 4.pdf]
